# Impact of Media-Induced Uncertainty on Mental Health: Narrative-Based Perspective

**DOI:** 10.2196/68640

**Published:** 2025-06-13

**Authors:** Ladislav Kesner, Veronika Juríčková, Dominika Grygarová, Jiří Horáček

**Affiliations:** 1 Center for Advanced Studies of Brain and Consciousness National Institute of Mental Health Klecany Czech Republic; 2 Faculty of Arts Masaryk University Brno Czech Republic; 3 First Faculty of Medicine Charles University Prague Czech Republic; 4 Third Faculty of Medicine Charles University Prague Czech Republic

**Keywords:** anxiety, cognitive psychology, social context, stress, uncertainty, media news

## Abstract

People worldwide are confronted with environmental and sociopolitical stressors that act as potent sources of subjective uncertainty. The uncertainty arising in response to the volatility and unpredictability of adversities is amplified by their representation or misrepresentation in media news. While the causal effect of media news on vicarious traumatization has been well established, we argue that the impact of negative media news is principally related to distress and anxiety stemming from the uncertainty-inducing effect of media representations of the state of the world. As a growing body of research suggests, minimizing uncertainty related to global stressors is a significant driver of media news use. However, extensive media exposure perpetuates stress and is associated with symptoms of psychopathology. The self-perpetuating vicious circle of worry and excessive media consumption has been amply confirmed by new research related to the COVID-19 pandemic. Furthermore, attempts to alleviate stress and anxiety stemming from uncertainties often result in maladaptive strategies. In particular, the adoption of rigid behavioral patterns may prompt various forms of socially detrimental behavior. Critical factors in prevention and remediation include limiting media overexposure and implementing therapeutic interventions that focus on increasing tolerance to uncertainty.

## Introduction

### Background

It has long been recognized that exposure to a stream of negative media news is detrimental to overall well-being and mental health. Although media news can have positive, neutral, or negative content, most news items have a negative valence [1,2]. It is well established that news consumers pay more attention to negative news [3], which also elicits stronger physiological and psychological reactions [4,5]. Negativity drives particularly online news consumption [6,7] and social media platform use [7]. Moreover, even people who do not receive regular news updates can still be confronted by news events through the people they follow on social media [8].

A large body of previous research has convincingly established the association between media exposure and symptoms of posttraumatic stress related to various life-threatening stressors, such as war conflicts [9,10], terrorism and other acts of mass violence [11-15], natural disasters [16,17], and recently also the COVID-19 pandemic (refer to "Uncertainty as a Defining Feature of the COVID-19 Pandemic" section). Additional lines of research have associated negative or violent news watching with anxiety and depression, self-destructive behaviors, and other mental health issues [18,19]. In addition to posttraumatic stress disorder (PTSD), anxiety and depression also showed significant linkages with indirect (media-transmitted) contact with disasters and large-scale violence [20,21].

A major implication of this research is that the long-standing dichotomy of *direct* versus *indirect* (ie, media-transmitted) exposure to psychological and traumatic stressors, as also codified in the *Diagnostic and Statistical Manual of Mental Disorders, Fifth Edition* [22], is problematic, as the distal and indirect levels are increasingly merged with the proximate and direct level of stressful impact. Recent research provided extensive evidence that indirect exposure is associated with stress symptoms and other psychopathologies comparable to those linked to direct exposure [13,16,23-27].

However, traumatic news constitutes only a minority of news items traversing various media outlets. While relatively scant, emerging evidence suggests that exposure to everyday nontraumatic news affects emotional states and mental well-being as well [28,29]. The dominant focus on vicarious psychological trauma may have obscured the fact that the complex relationship between media news consumption and its negative mental health effects appears to be, to a large extent, mediated by uncertainty. In this narrative-based perspective, we present a sustained argument that uncertainty now must be recognized, alongside vicarious traumatization, as a major outcome of media-transmitted adversities. First, we focus on reviewing current research linking media consumption with current sociopolitical and environmental adversities. Next, we zoom in on cognitive mechanisms through which media news elicits uncertainty and negative anticipation before turning to behavioral consequences of media-related uncertainty. Finally, we conclude with a brief section on remediation and resilience building.

### Global Adversities, Uncertainty, and Media

Uncertainty is a multidimensional construct and has been defined in various ways [30-32]. At its most basic, uncertainty is a subjective state that captures a person’s belief about the state of the world. It is often used to refer to the lack or inconsistency of information about an event or situation. Computations of subjective estimates of uncertainty predict acute stress responses [33] and depressive symptoms [34] in humans. Several prominent models posit a direct causal link between uncertainty and anxiety [35-37]. Indeed, anxiety has been defined as “anticipatory affective, cognitive, and behavioral changes in response to uncertainty about a potential future threat” [34]. Furthermore, subjective feelings of uncertainty, or uncertainty distress [38], may be accompanied by or instigate a host of other mental states, such as helplessness, frustration, anger, guilt, and grief [39].

Currently, there are 2 major factors mediating the intensity of the psychological impact of uncertainty on populations. First, global communities are confronted with a plethora of interconnected sociopolitical and environmental adversities—all characterized by high levels of complexity, unpredictability, and volatility—from progressing and potentially catastrophic climate change, accelerating economic inequality, wars and conflicts, widespread displacement and migration, the resurgence of authoritarian political tendencies in many parts of the world to the COVID-19 pandemic and potential threats of uncontrolled artificial intelligence, to name but the most obvious ones. Other chronic problems arising from more local contexts add to the burden of global adversities. While at present there is no generally accepted definition of what constitutes a “stressful event” [40,41], it is nevertheless evident that people are confronted with a range of *psychological* and *traumatic* stressors [42]. To varying degrees, they span ongoing situations as well as expected or potential future developments. The protracted, inherently complex, and unpredictable nature of such adversities translates into numerous *situational uncertainties* about personal prospects and the possibility of achieving life goals [43,44]. Uncertainty-related worry and anticipation range from severe existential threats, including the possibility of imminent death (Will the Russian attack on Ukraine escalate into a nuclear war?) and threats to the achievement of primary life goals and motivations to less severe but still worrisome and potentially traumatic uncertainties regarding social and economic security. While some may have a limited duration (Will the feared politician be elected in the upcoming election?), many are ongoing with no clear resolution in sight. For many people, these global stressors will additionally exacerbate personal uncertainties, involving doubts in self-views and worldviews [45].

Uncertainty most often has a temporal dimension, as it is related to outcomes that are supposed to occur in the future and stems from the person’s inability to predict future events. The inherently anticipatory nature of uncertainty-related stressors and their negative consequences on mental health have been long recognized [46,47]. In the realm of sociopolitical adversities, uncertainty and anticipation of economic shocks, not just their occurrence, were highlighted as a significant causal mechanism linking poverty and mental illness [48]. Studies have demonstrated that uncertainty surrounding elections, governance changes, and economic downturns can lead to future-oriented anxiety, heightened vigilance, and cognitive rigidity [49]. Therefore, it is not surprising that a recent psychological survey showed that 81% of US adults pointed “global uncertainty” as a source of chronic stress [50]. Chronic uncertainty exerts a particularly heavy toll on people living under multiple strains and in precarious conditions, such as refugees and displaced persons [51-53], and on people living under *continuous traumatic situations* intensively covered by the media, such as populations affected by Israeli-Arab conflicts or the war in Ukraine. Individuals living in such chronic situations seem to be mainly concerned with the future, that is, with anticipated future danger and threat, and their thinking is dominated by fantasies of what might happen and ways of avoiding it [54,55].

Second, as we further elaborate in the following sections, media representations or misrepresentations of the reality and potential consequences of adversities further exacerbate uncertainty, along with feelings of uncontrollability and unpredictability, as well as negative anticipation toward socioenvironmental stressors. If theoretical models of uncertainty, particularly Bayesian computational models, posit the uncertainty to arise from discrepancies between top-down expectations and sensory outcomes [56,57], it bears emphasizing that the sociopolitical and environmental adversities are to a large extent (or to many people) hidden to direct sensory observation, their reality and potential consequences being established only by representations diffused by mass and social media. Another key factor in sociopolitical uncertainty is the role of misinformation and propaganda, which contributes to fear, panic, and distrust, thereby exacerbating psychological distress. Misinformation and propaganda have been linked to increased polarization and cognitive rigidity [58], the adoption of conspiracy theories as a means of reducing uncertainty [59], and heightened distress and anxiety due to conflicting narratives and media sensationalism [53].

## Two Case Studies

### Uncertainty as a Defining Feature of the COVID-19 Pandemic

Pervasive uncertainty has been a fundamental aspect of the public health crisis caused by the COVID-19 pandemic—a global and chronic psychological stressor with severe implications for mental health and well-being [60,61]. The COVID-19 pandemic provided new incentives for a reconceptualization of the very notion of the traumatic stressor (as captured, eg, in *Diagnostic and Statistical Manual of Mental Disorders, Fifth Edition*), as its negative impact relates to the future rather than the past and to indirect as much as to direct exposure [62,63]. While this reconceptualization is theoretically compelling, it remains underexplored empirically and requires further research to assess its validity in relation to long-term diagnostic outcomes.

Robust evidence from extensive research, including both cross-sectional and longitudinal designs, using both convenient and representative samples, confirms that excessive exposure to pandemic-related media is a substantial predictor of detrimental psychological effects. These effects include heightened levels of anxiety [64], depression [64,65], psychological distress [66,67], posttraumatic stress symptoms [68], schizotypal traits [69], and substance use [70].

This negative impact on the psychological state has been found to be more pronounced in social media than in traditional media [64,68,71-73]. Several studies have focused primarily on the use of online media or social media during pandemics. They confirm a connection between excessive electronic (internet) media exposure and the prevalence of stress, generalized anxiety, depression, and long-term psychological distress [66,70,74]. Such a pattern was confirmed in a study using ecological momentary assessments that found a positive association between daily exposure to COVID-19 news, worry, and hopelessness [75]. Social media has also fueled the rapid spread of misinformation and rumors, which can create a sense of panic and confusion among the public [76], underscoring the importance of combating the “infodemic” during a public health emergency [77]. However, these studies rarely distinguish between specific platforms (eg, Twitter [now rebranded as X], Facebook, Telegram, TikTok, and Reddit) or types of use (eg, news seeking vs social interaction). This is a significant limitation, as different platforms foster distinct emotional climates and are shaped by unique algorithmic dynamics, which may influence their psychological impact in different ways.

While the existing body of literature robustly supports a link between media exposure and adverse psychological outcomes during the COVID-19 pandemic, it is important to note that most of these studies rely on cross-sectional designs and self-report measures. Therefore, our understanding of the reciprocal relationship between distress and media use—such as whether psychological distress drives increased “doomscrolling” or vice versa—remains limited. However, some studies have also used more rigorous methodologies, including longitudinal designs and ecological momentary assessment [75]. In addition, there is a lack of standardization in how media exposure is defined and measured, with few studies differentiating between media platforms, content types, or modes of engagement [71,73]. There is also a notable lack of research into potential protective factors, although some studies have begun to address this gap [71].

### Current War in Ukraine

War-induced uncertainty encompasses a wide range of anxieties, including geopolitical instability (eg, fear of war escalation, nuclear threats, and global security risks); economic uncertainty (eg, rising energy prices, disrupted supply chains, and inflation concerns); and humanitarian crises, such as displacement, refugee movements, and ethical dilemmas regarding international response. Individuals with high levels of intolerance of uncertainty (IU) are more likely to experience heightened levels of stress when confronted with uncertain or unpredictable circumstances, such as wartime [78]. Fear of war has been found to increase the level of stress, anxiety, and depression, while also strengthening the future anxiety and IU [79]. The anticipatory uncertainty surrounding the conflict (eg, potential escalation, economic instability, and geopolitical tensions) parallels the uncertainty associated with global health crises but differs in that it involves military aggression, political narratives, and humanitarian crises, which create unique patterns of stress and information-seeking behavior [53]. Just as with the COVID-19 pandemic, social media plays a dual role in war-related uncertainty—it serves as both a source of information and a catalyst for misinformation and emotional distress. Real-time updates create a heightened sense of crisis, leading to compulsive checking of news feeds (doomscrolling), a behavior that has been directly linked to increased psychological distress and rumination [80].

The ongoing war in Ukraine provides a critical example of how media coverage of armed conflicts can amplify uncertainty-related mental health symptoms, even in populations not directly involved in the conflict. The pervasive dissemination of war-related news through various media channels has profound implications for public mental health. Individuals, even those geographically distant from conflict zones, can experience significant psychological distress upon exposure to such content. Recent research highlights that war-related uncertainty, particularly media-induced exposure to conflict, has significantly augmented levels of stress, anxiety, depression, and posttraumatic stress in Czech, Slovak, German, Polish, Italian, and even Taiwanese populations [55,81-83].

Kalaitzaki et al [84] provided a cross-sectional analysis of the impact of war across 11 countries on populations in areas directly or indirectly affected by the conflict, focusing on war-related stressors and PTSD symptoms. Individuals exposed to war-related stressors, including displacement, violence, and loss, are at a heightened risk for developing PTSD. However, the authors also highlight the significant role of media coverage and uncertainty, which are not often directly linked to conflict but have substantial psychological effects. Continuous media coverage, especially news reports focusing on graphic images or uncertain political developments, was found to heighten feelings of anxiety and helplessness among those already traumatized by the war. Constant media bombardment, especially when coupled with the prolonged uncertainty about the war’s resolution, contributes significantly to the mental health burden experienced by affected populations. Uncertainty, in this case, is not just related to the physical dangers of war but also to the unpredictability of the future and a general sense of destabilization. This research emphasizes that the mental health effects of war extend beyond direct trauma, with media and uncertainty being central factors in the broader psychological impact of conflict.

## Individual Differences in IU

The ability to tolerate uncertainty is a critical factor in adapting to unfavorably changing conditions with low predictability. Conversely, the IU has been defined as “an individual’s dispositional incapacity to endure the aversive response triggered by the perceived absence of salient key, or sufficient information, and sustained by the associated perception of uncertainty” [32]. IU, as a set of negative cognitive, affective, and behavioral reactions to situational uncertainty [38,85], predicts acute subjective and psychological stress response in humans [33] and defensive responding. It functions as a catalyst of anticipatory anxiety and catastrophic thoughts and negative rumination on possible negative and catastrophic outcomes [32,86]. IU has been identified as a critical transdiagnostic risk factor and component of internalizing psychopathology across a range of mental disorders, such as generalized panic and social anxiety disorders, obsessive-compulsive disorder, depression, eating disorders, and others [87-89]. Individuals with high levels of IU are more likely to experience heightened levels of stress when confronted with uncertain or unpredictable circumstances, such as wartime [78]. This connection has been amply documented in the context of COVID-19: recent research has confirmed a direct association between IU and negative mental health outcomes during the pandemic [90-94].

A number of recent studies have specifically examined the link between media use, IU, and negative psychological effects [68,95-97]. A direct effect of IU on problematic social media use [98] and mobile addiction [99], as well as moderation effects between both depression and anxiety and problematic social media use, was found [100]. IU also mediated the effect of fatigue related to COVID-19 on depressive symptoms [101] and partially mediated the relationship between media exposure to COVID-19 and acute stress [102].

## Demographic Differences in IU

Emerging research indicates that IU varies across individuals and populations, influenced by demographic factors such as age, gender, cultural background, and socioeconomic status. While individual differences in IU significantly influence responses to media-induced uncertainty, understanding demographic differences in IU is crucial for developing targeted therapeutic implications for interventions aimed at reducing media-induced stress. Research indicates that IU levels fluctuate across the lifespan [103]. A recent meta-analysis revealed significant age-related differences, with adolescents exhibiting higher IU compared to adults and older adults [104]. This heightened intolerance in younger individuals may be attributed to developmental factors and varying coping mechanisms. In addition, studies suggest that IU correlates with anxiety and other psychological issues in older adults.

Regarding media use, research has shown that younger populations (aged between 15 and 20 years) tend to engage in higher levels of media consumption, making them more vulnerable to doomscrolling and increased anxiety [105]. Older adults (aged between 45 and 55 years) are more susceptible to economic and political uncertainty, exhibiting stress responses driven by financial instability and governance concerns [106]. Therefore, for younger individuals, strategies such as limited media consumption, cognitive restructuring, and digital literacy training may be particularly effective. In contrast, older adults may benefit from mindfulness-based interventions (MBIs) and cognitive reframing techniques to mitigate uncertainty-related stress [107].

Gender has been identified as another significant factor influencing IU. Studies indicate that women often exhibit higher levels of IU [108] and score higher in constructs related to health anxiety and IU [109,110], showing a stronger relationship between IU and negative future orientation. Research involving university students found that female participants scored higher on measures of trait worry, IU, and cognitive avoidance compared to male participants. While both male and female entrepreneurs face uncertainty, their decision-making processes may differ [111]. However, findings on how IU and gender jointly affect decision-making strategies such as effectuation and causation are mixed, indicating the need for further exploration. These findings suggest that women may experience and react to uncertainty differently than men, potentially due to a combination of biological, psychological, and sociocultural factors.

However, it is important to note that some studies report no significant gender differences in IU [112]. For example, research investigating the relationship between IU and psychological well-being found no gender differences in IU scores among participants. This suggests that the impact of IU may be more pronounced in certain contexts or populations rather than being universally higher in women.

Socioeconomic status and education are other crucial factors that can influence an individual’s IU. While specific studies on the direct relationship between socioeconomic status and IU are limited, it is plausible that individuals with higher socioeconomic status and education levels may develop more effective coping strategies for dealing with uncertainty. Higher socioeconomic status individuals often have access to better resources, health care, and social support systems, which can buffer the negative effects of uncertainty and stress. Conversely, individuals from lower socioeconomic backgrounds may face additional stressors, such as financial instability, limited access to health care, and social discrimination, which can exacerbate IU. These stressors may increase their vulnerability to anxiety and related disorders as the uncertainty surrounding their daily lives becomes more pronounced. The chronic stress associated with economic hardship can lead to heightened sensitivity to uncertainty, making it more difficult to cope with ambiguous situations.

In addition, educational attainment plays a role in shaping an individual’s ability to manage uncertainty. Higher levels of education are associated with better problem-solving skills, greater self-efficacy, and improved emotional regulation, all of which can reduce the impact of IU [113]. In contrast, individuals with lower educational attainment may lack these coping resources, making them more susceptible to the negative psychological effects of uncertainty [114].

Cultural influences are another important demographic consideration. IU is not only shaped by individual psychological traits but also by the social and cultural environments in which people live, and cultural factors significantly influence how individuals experience and cope with IU. In collectivist cultures, where social harmony, group cohesion, and interdependence are highly valued, uncertainty may be viewed as a potential threat to relational stability. Therefore, individuals from collectivist cultures might experience heightened anxiety and distress when faced with uncertain social outcomes or the possibility of disrupting group expectations [115]. This socially driven uncertainty can increase IU, particularly in the context of interpersonal relationships and social approval [116]. By contrast, in individualistic cultures, where autonomy, independence, and self-expression are prioritized, uncertainty may be experienced as a threat to personal control and self-efficacy [117]. Individuals in these cultures may experience greater distress when uncertainty undermines their sense of personal agency or the achievement of individual goals [118]. The difference in how uncertainty is perceived—social versus personal control—suggests that therapeutic approaches should be tailored to cultural contexts to better address the distinct ways in which IU manifests.

## Mediating Role of Vigilance and Future-Oriented Thought

Psychological and neural mechanisms through which uncertainty-inducing stressors elicit negative mental states, particularly anxiety, and may lead to psychopathology have been extensively described in the literature [31,35,36,57,119,120], and these models are broadly relevant in the present context. Due to space constraints, we briefly focus on 2 processes that we consider critically important for how media news is processed: vigilance and future-oriented thought.

### Vigilance

Humans display sustained vigilance and defensive responses under conditions of uncertainty [31,32,85]. While sustained vigilance represents the initial adaptive response under conditions of uncertainty [35,120], it can develop into hypervigilance, that is, increased attention to threatening or potentially threatening stimuli. Hypervigilance has been singled out as 1 of 5 key processes involved in aberrant and excessive anticipatory responding to uncertainty [31]. It is tightly coupled with attentional threat bias, referring to the fact that negative valence content captures human attention more than other content [121,122]. Converging evidence indicates that negative emotional content is prioritized over neutral content even during early stages of visual attention [122-124]. Uncertainty-related hypervigilance “locks into” the aforementioned propensity of news to prioritize content with negative valence and the tendency of people in a situation of high uncertainty to explore negative information [125] and appraise emotionally ambiguous information in negative terms [126], thus initiating a vicious circle, which we describe in the subsequent sections.

Although the mechanism of hypervigilant response to uncertain threat is well supported in the literature, it has not been investigated in studies that would accurately reflect real-world media consumption patterns and that could account for the complex interplay between the type of media consumed and the individual’s baseline level of anxiety or stress. Thus, while uncertainty-related vigilance may align with news media’s tendency to emphasize negative content, further research is needed to explore how the framing of news, individual differences, and media platform type contribute to this relationship.

### Future-Oriented Thought

As uncertainty-related worry mostly concerns future events and situations with an uncertain outcome [127], the aversive uncertainty generated by the appraisal process of media news hinges on the person’s mental construction of some possible future state of affairs implied by the news item. The key cognitive mechanisms are episodic and semantic simulations of future events [128]. Episodic simulation constructs a detailed mental representation of a specific autobiographical situation in the future [129]. Much less is known about semantic simulation–driven expectations, that is, predictions and future thinking about more general or abstract states of the world that may arise in the future (eg, thinking about what the consequences of global warming will be like 25 years from now) [130]. Both forms are likely involved and mutually intertwined in the appraisal process of media-related stressors. As imagining aversive events has emotionally aversive consequences, internal simulations themselves incur some of the same costs as real-world experience [131]. Growing literature connects psychological distress, particularly internalizing psychopathology, to disturbances in both episodic and semantic future thinking. It has long been recognized that future-oriented cognitions play a central role in the development of depression and anxiety [132,133]. Both conditions are commonly characterized by negative, future-oriented thought patterns [134,135]. Mental simulation of future events may be the mechanistic link between uncertainty (as a breeding ground of anxiety) and affective responses [136].

Although both types of future-oriented thinking are central to the appraisal of uncertainty, the literature has largely focused on episodic simulations, with limited research on the role of semantic simulations in the context of media-induced stress. Notably, while there is growing evidence that future-oriented thinking can both exacerbate and mitigate psychological distress, the findings are inconsistent. For example, some studies suggest that structured mental simulations of stressful events can improve emotional regulation and increase the propensity for problem-focused coping [137,138]. In contrast, other studies argue that suppressing the mental simulation of feared future events can help prevent the onset of anxiety [139].

Furthermore, much of the existing research predominantly focuses on clinical populations, with little attention paid to how future-oriented cognition operates in nonclinical groups. This is a significant gap in the literature, as many individuals in the general population experience uncertainty-related anxiety without meeting the clinical criteria for disorders such as generalized anxiety or depression. A deeper exploration of the neural mechanisms underlying future-oriented thought in nonclinical populations, particularly in response to uncertainty-inducing media, is an important avenue for future research.

In summary, the existing literature on vigilance, future-oriented thought, and their roles in media-induced anxiety has provided important insights. However, significant gaps still remain that must be addressed. Future research should consider more longitudinal and experimental designs to establish causal relationships, explore the effects of different media types, and investigate the neural mechanisms involved. In addition, expanding the focus beyond clinical populations will help clarify how these processes operate in the general population and contribute to the development of anxiety and other mental health issues. By addressing these gaps, we can enhance our understanding of how uncertainty-related processes interact with media consumption to shape psychological and emotional responses.

## Behavioral Consequences of Subjectively Felt Uncertainty

### Vicious Circle of Uncertainty and Media News Consumption

As Hirsh et al [35] aptly observe, much of our lives are spent trying to reduce and manage uncertainty. Management of uncertainty is a complex process, and reduction or resolution are not the only strategies people pursue in the face of uncertainty [140]. Nevertheless, aversive feelings stemming from uncertainty typically lead to attempts to minimize them, which often involve maladaptive responses such as hypervigilance, repetitive negative thinking, reassurance seeking, and increased checking behavior. Uncertainty reduction theory [141] posits that people are motivated to seek information in the aftermath of a traumatic or threatening event to reduce anxiety.

As discussed earlier, a substantial body of research indeed confirms that people in uncertain situations, such as disaster events or pandemics, seek information via media to alleviate negative feelings, yet extensive media exposure perpetuates stress and is associated with symptoms of psychopathology, and this pattern is particularly characteristic of the use of social media [27,74,142-145]. Conversely, news avoidance can have a positive effect on mental well-being [146]. While some degree of media use to obtain information and alleviate distress is adaptive in the setting of momentary or long-term stressors, in practice, there is no clear-cut division between adaptive information seeking, which (at least initially) may serve to guide rational decision-making and action vis-à-vis particular adversity, and compulsive news consumption, mainly driven by the need to alleviate aversive feelings of worry and anxiety. A need to minimize uncertainty is thus a major driver of news use, which, however, may escalate into a vicious circle of iatrogenic-like media consumption, which reciprocally further increases worry and anxiety.

A growing number of studies currently provide empirical evidence of this pattern. In the study conducted in the aftermath of the 9/11 attacks, Cho et al [147] established that negative affect resulting from television news use after the attacks drove future television news use and predicted negative affect responses several months later. Such a pattern has been confirmed in a recent longitudinal study following 2 terrorist attacks: trauma-related media exposure perpetuates a cycle of high distress and worry, leading to increased subsequent trauma-related media consumption, which further promotes increased distress [143]. In a similar way, a study of anticipatory media exposure to hurricanes found that personally forecasted posttraumatic responses and storm-related media consumption before an approaching hurricane are important correlates of poststorm psychological adjustment [148].

The self-perpetuating vicious circle of worry and excessive media consumption has been again amply confirmed by new research related to the COVID-19 pandemic. Several studies found that worry about the pandemic predicted increased consumption of pandemic-related media and doomscrolling, which in turn led to further increases in negative emotions and pandemic-related worry [74,149-151]. According to daily survey studies, increased exposure to COVID-19 news on one day increased COVID-19 worries the next day, and higher worries on one day predicted higher media exposure the next day [150,151]. Increased media consumption during the pandemic may thus initially serve as a potential coping strategy to handle pandemic-related distress but often results in inducing more worries through mechanisms of overexposure and rumination [149]. Individuals with high levels of trait anxiety, neuroticism, and a history of severe maltreatment, as well as those with more severe baseline psychopathology, tend to exhibit increased overall levels of media exposure and thus constitute a group considered particularly vulnerable [72,150]. Moreover, the inherent design of contemporary media platforms tends to amplify these effects. Previous research has also established that increases in IU correlate positively with mobile phone penetration and internet use. These technologies thus increase reassurance seeking and reduce spontaneous, everyday exposures to uncertainty, which may lead to psychopathology by increasing IU and anxiety [152]. Research shows that individuals with higher IU who frequently searched the internet for COVID-19 information experienced greater fear of the virus—a fear that is both a consequence and a driver of IU [95].

### Cognitive Inflexibility, Compensatory Control, and Tightening of Beliefs

In addition to information seeking and fact-checking, people experiencing uncertainty-related worries and anxiety often engage in processes of compensatory control in an effort to imbue the world with order and predictability [59]. Uncertainty and uncontrollability breed the loss of meaning. The aversive reactions then result both from the perceived threat to one’s motivations and goals and from the decreasing ability to make sense of the changing and volatile social environment and one’s prospects in it, which prompts maladaptive strategies to restore the sense of meaning to events in the world. A drive to reduce uncertainty is tightly coupled with cognitive inflexibility. Individuals with a lower threshold for tolerating uncertainty tend to be more rigid and closed-minded in their worldviews, values, and attitudes [45,153], especially when uncertainty is explicitly linked with threat [154,155]. Psychological models, such as the uncertainty-identity theory [156], meaning maintenance model [157], and compensatory control theory [158,159], have elaborated on how the defensive need to alleviate uncertainty leads to group identification, ideological inflexibility, and dogmatism. Such “tightening of beliefs” functions as a defensive response to intolerable stress and uncertainty and may affect mental health in a transdiagnostic manner [160]. The “tight beliefs” or rigid interpretations may be linked to conspiracy thinking [58] and may attain a form of delusional ideation [44]. They have profound political and social consequences as they fuel polarization, partisanship, fanaticism, and extremism [161,162]. Ultimately, such tight beliefs contribute to the spreading of conspiracy theories and other forms of socially detrimental behavior, thus further enhancing subjective uncertainties about the state of the world.

However, attempts to minimize short-term uncertainty by adoption of rigid cognitive structures and behavioral patterns are, in the long run, often maladaptive strategies [32,35]. Typically, people who turn to conspiracy beliefs in an attempt to alleviate the negative experience of uncertainty do not succeed in this attempt and may even experience short-term increases in uncertainty aversion, anxiety, and existential dread [163]. Importantly, a tightening of beliefs under conditions of chronic stress and uncertainty does not automatically translate into maladaptive or deviant social behavior.

## Strength of Evidence and Methodological Considerations

While, as reviewed, there is growing research linking IU to media consumption and psychological discomfort, many of these studies rely primarily on cross-sectional designs and self-reported measures, which have inherent limitations. Cross-sectional studies only record 1 time point, making it impossible to discern causality—whether IU causes higher media use and anxiety or whether excessive media exposure worsens IU and distress. Furthermore, self-report measures are susceptible to recall bias and social desirability effects, as individuals may overestimate or underestimate their media intake and emotional impact [65]. Future research should prioritize longitudinal designs and experimental methods to elucidate the causal relationships between media exposure, IU, and psychological distress. The measurement of media exposure is often poorly standardized, ranging from self-reported hours to vague categories such as “many times per day,” and rarely differentiating between active and passive use or the specific type of content consumed. Many studies rely on self-reported data, which may be subject to recall bias, as participants often struggle to accurately estimate their media consumption habits [76]. Future studies should incorporate objective digital tracking methodologies to refine these findings and better assess real-time media exposure patterns.

Another important limitation is the lack of consistent definitions and measurements of IU across studies. Some studies measure IU using general anxiety measures, while others use particular IU scales to detect anticipatory worry or avoidance actions. This inconsistency makes it difficult to evaluate results across populations and situations [32].

## Potential Biases in Literature

Many studies rely on convenience samples, often recruited through online platforms, which raises concerns about sampling bias—such as the overrepresentation of the young and educated, with digital literacy—and limits the generalizability of the findings. A related problem is the overrepresentation of western, educated, industrialized, rich, and democratic populations in psychology research [164]. Most studies on IU, stress, and media consumption have been conducted in North America and Western Europe, which limits the cross-cultural application of the findings. For example, while American and European populations report high levels of uncertainty-induced stress due to political instability and economic downturns, research from East Asia suggests that collectivist cultures may use different coping mechanisms, such as social cohesion and community-based stress buffering [165]. In contrast, extended uncertainty is frequently normalized in Middle Eastern or conflict-affected areas, resulting in distinct patterns of emotional regulation and resilience [53].

## Cross-Cultural Perspectives on Media Influence

The media’s influence on generating uncertainty perceptions differs around the globe. While Western media regularly sensationalizes crises and political instability, resulting in fear-driven engagement (doomscrolling), authoritarian media frequently restricts uncertainty-inducing news to maintain societal control. For example, during the COVID-19 pandemic, studies found that media exposure increased uncertainty-related anxiety in democratic countries, whereas in China, state-controlled media reduced coverage of negative outcomes, resulting in lower reported stress levels despite real risks [102]. Such disparities imply that media-induced doubt is influenced not only by global crises but also by governmental policies, media freedom, and cultural attitudes toward uncertainty.

## Preventive Measures and Remediation

### Managing Media Consumption

Even a cursory search of the internet yields dozens of popularizing articles from mental health professionals advising readers to limit the intake of negative media and avoid excessive doomscrolling and compulsive social media use for the sake of their mental health. Behavioral coping with the influx of negative media news must be primarily focused on the aforementioned amplifying effects of the media in spreading uncertainty and stress, along with their adverse mental states. However, while behavioral coping strategies must address the amplifying effects of media in spreading uncertainty and stress, a nuanced approach is needed, as complete avoidance may also have unintended consequences.

As noted earlier, robust evidence has shown a positive association between the frequency and total time spent watching or reading media news and symptoms of mental distress, highlighting the need to limit media exposure [10,64,66,166]. Even ≥1 hours of daily television watching might have some negative consequences [10,13,167]. Specifically, Bendau et al [64] reported that the critical threshold of 7 times per day and 2.5 hours of media exposure differentiates between mild and moderate symptoms of anxiety and depression. This implies that while avoidance is generally considered a less adaptive strategy, in the present context, what might be termed “informed avoidance” might prove to be a positive reaction. Preventing overexposure to media, particularly in groups considered vulnerable, is thus critical to buffering distress.

The specific circumstances of media consumption present another opportunity for intervention. There is some evidence to suggest that negative emotional responses to negative news can be mitigated by constructive news reporting and peer discussion [168]. Patterns of reading and watching may also play a role, with reading entire articles presenting a safer alternative to mere headline browsing [71]. However, reading anonymous and often highly negatively balanced comments under web news articles leads to an increase of subjectively perceived stress, anxiety, and depression induced by the media [71]. Moreover, in the absence or ambiguity of information, people contribute to the spread of misinformation [169] or disinformation, a subject of much recent attention [170-172]. Adaptive media consumption patterns must include a critical mindset when consuming media, question potential motivations behind the presented information, and avoid untrustworthy sources of news content and exposure to disinformation or fake news.

Consequently, interventions should incorporate media literacy training, equipping individuals with critical thinking skills to evaluate sources, assess potential biases, and recognize disinformation patterns [173]. Cross-cultural studies suggest that individuals in societies with high levels of trust in institutions and regulated media environments (eg, Nordic countries) exhibit greater resilience to misinformation-related stress compared to those in highly polarized or authoritarian settings, where media manipulation is prevalent.

### Promoting Tolerance to Uncertainty and Cognitive Flexibility

Given the crucial role of uncertainty in the cascade of negative effects of media exposure and propaganda, increasing tolerance of uncertainty is a critical goal of intervention. Cognitive behavioral therapy for IU (CBT-IU) [174] is a specialized form of cognitive behavioral therapy (CBT) designed to help individuals develop adaptive responses to uncertain situations [175]. While originally developed for anxiety disorders, CBT-IU has been successfully applied in contexts such as pandemic-related stress, economic instability, and geopolitical crises [38]. However, many studies examining CBT-IU effectiveness rely on short-term intervention periods, making it difficult to assess long-term outcomes. More research is needed to evaluate sustained behavioral changes in uncertainty management strategies. Beyond traditional CBT, *third-wave* behavioral therapies, including acceptance and commitment therapy (ACT), dialectical behavior therapy (DBT), metacognitive therapy, and mindfulness-based cognitive therapy [176,177], offer promising avenues for increasing resilience to uncertainty [176]. These approaches emphasize acceptance-based strategies rather than avoidance, enabling individuals to engage with distressing thoughts without excessive reactivity.

ACT or DBT generally share an emphasis on acceptance and mindfulness, encourage individuals to accept their emotions and thoughts, and can help individuals to better manage their emotions when confronted with distressing media content.

Another approach, metacognitive therapy, aims to change unhelpful thought patterns related to rumination, worry, and cognitive processes that can lead to distress.

Recent studies suggest that ACT interventions significantly reduced anxiety symptoms during the COVID-19 pandemic, particularly among adolescents and health care workers, who faced high levels of prolonged uncertainty [178]. Several studies demonstrated that ACT-based interventions led to significant reductions in depression and stress symptoms in pandemic-exposed populations, with long-term effects persisting months after initial treatment [179]. However, it is important to acknowledge cultural variability in treatment efficacy—while ACT and DBT have been widely implemented in Western populations, studies examining their effectiveness in collectivist cultures remain limited, warranting further cross-cultural validation [180].

MBIs represent another promising approach for enhancing IU and improving emotional regulation. Empirical evidence points out that mindfulness-based approaches may improve stress tolerance and coping [181]. Mindfulness-based stress reduction programs have been shown to ameliorate symptoms of anxiety, depression, and emotional dysregulation during global crises [182]. MBIs help individuals develop a nonreactive awareness of distressing content, breaking the cycle of negative rumination triggered by media exposure [183]. Mindfulness was instrumental in developing the capacity to disengage from attention capture by future- or past-oriented thinking, thus ameliorating mental symptomatology related to uncertainty and the loss of pandemic situations [184]. The MBSR program successfully reduced stress and anxiety while improving emotion regulation in participants during highly stressful conditions caused by the global pandemic [185].

All these preventive and remediation interventions, to varying degrees, foster neuroplasticity-related adaptations in brain regions involved in emotional regulation and cognitive flexibility [186-188]. Functional neuroimaging studies indicate that adaptive coping with uncertainty is associated with alterations in the perigenual anterior cingulate cortex and ventromedial prefrontal cortex [187], regions implicated in resilience to stress [189]. However, more research is needed to determine how specific mindfulness interventions influence neurobiological pathways associated with uncertainty regulation across different populations.

Moreover, there is empirical evidence that exposure therapy can be an effective treatment for people with generalized anxiety disorder, citing specifically in vivo exposure therapy (exposure through a real-life situation [190]), which has greater effectiveness than imaginal exposure in regard to generalized anxiety disorder. The aim of in vivo exposure treatment is to promote emotional regulation using systematic and controlled therapeutic exposure to traumatic stimuli [191]. Behavioral exposure involves gradually confronting feared situations or uncertainties in a controlled manner. This technique helps individuals build resilience and reduce avoidance behaviors associated with IU. Over time, exposure can lead to habituation and a decrease in anxiety responses to uncertain situations.

The effective prevention and remediation strategies for media-induced uncertainty stress require a multilevel approach that integrates adaptive media consumption, cognitive restructuring, and neuroplasticity-informed resilience training. Given the increasing prevalence of global crises and uncertainty-inducing media narratives, interventions aimed at enhancing cognitive flexibility, promoting mindfulness, and developing critical media literacy are essential for mitigating the negative psychological consequences of excessive media exposure.

### Differences in Remediation Strategies According to Demographic Factors

In addition to the existing preventive and remediation strategies, it is crucial to consider demographic variations in media consumption patterns and the corresponding responses to media-induced uncertainty. Research highlights that different age groups exhibit significant differences in media use, emotional responses to media content, and their receptiveness to various therapeutic interventions. For example, younger generations, particularly those aged between 15 and 20 years, are more likely to engage heavily with social media and digital platforms, making them particularly vulnerable to the effects of doomscrolling and compulsive media consumption [192,193]. Such media consumption behaviors are linked to increased anxiety, depression, and stress, making interventions that focus on reducing media exposure especially beneficial for this cohort [64,76]. Limiting excessive media use could mitigate these effects by reducing the amount of negative information consumed, as discussed in the context of “informed avoidance” [64].

In contrast, older generations, such as those aged between 45 and 55 years, may engage with media differently. They often rely more on traditional news outlets such as television and newspapers, as opposed to social media, which could lead to distinct emotional responses and stressors when confronted with global crises, such as the COVID-19 pandemic or the Ukraine war. In these populations, MBIs, including mindfulness-based cognitive therapy and ACT, have shown promise in helping individuals cope with stress and uncertainty [182,194,195]. Such interventions, which focus on emotional regulation, cognitive flexibility, and acceptance, are particularly effective for older individuals who may struggle with rumination and excessive worry when faced with distressing news [178,179].

In addition to considering age-based distinctions, it is also essential to evaluate gender differences. Research has consistently shown that men and women exhibit different patterns of media consumption and varying emotional responses to distressing (media) content [196,197]. Women are more likely to engage with emotional and social media content, which often leads to heightened emotional distress in response to negative news [192]. Women also tend to engage in more social media use, including doomscrolling, as a coping mechanism for stress, which can exacerbate symptoms of anxiety and depression [198]. Therefore, interventions that focus on reducing media consumption may be particularly beneficial for women, as limiting exposure to negative news could help mitigate the psychological effects of distressing information [64]. The increased susceptibility of women to anxiety and depressive symptoms suggests that media avoidance strategies could be more effective for this group.

In contrast, men often exhibit different emotional responses to media content. Men may experience higher levels of anger and frustration when exposed to content that challenges their values or sense of control [197]. They are also generally less likely to seek help for mental health issues, which can influence how they cope with media-induced stress [199]. Therefore, men may benefit from therapeutic approaches that emphasize emotional regulation and acceptance, such as MBIs or ACT, which focus on managing distress without necessarily reducing media exposure [194,195].

Given that women tend to ruminate more in response to stress, MBIs targeting rumination and emotional regulation might be particularly helpful [200]. By contrast, men may benefit from ACT-based approaches, which encourage acceptance of difficult emotions and values-based actions, offering a framework that could be more in line with how men approach emotional and cognitive regulation [201]. Moreover, cultural influences significantly impact how individuals experience IU and underscore the importance of adapting therapeutic approaches to align better with cultural values and experiences. CBT, a common intervention for IU, can be adapted to these cultural contexts by integrating culturally relevant coping strategies that address the unique ways in which uncertainty is perceived and managed.

For instance, in collectivist societies, individuals may benefit from therapeutic interventions that focus on enhancing social support and community-based coping strategies, helping them navigate uncertainty in ways that align with their cultural values [116]. In contrast, in individualistic cultures, therapy may emphasize building self-efficacy, personal resilience, and cognitive restructuring to help individuals tolerate uncertainty without feeling a loss of control [118,202]. Individualized therapy models, such as CBT and ACT, which emphasize self-directed coping and personal resilience, are more commonly used [203].

MBIs also require cultural tailoring to enhance acceptance and effectiveness. For example, in cultures with strong spiritual or religious traditions, integrating culturally relevant mindfulness techniques can improve therapeutic outcomes [204]. Research has demonstrated that culturally adapted MBIs, such as modifications to MBSR programs, have enhanced effectiveness in ethnic and Indigenous populations by incorporating community-centered healing practices [205].

Furthermore, cultural competence in therapy plays a vital role in managing IU. Approaches such as intercultural therapy emphasize an understanding of the client’s cultural background, values, and beliefs to optimize treatment outcomes [203]. Studies suggest that culturally tailored therapy is more effective in addressing uncertainty-related distress, as it aligns therapeutic techniques with cultural worldviews and coping mechanisms [117]. Thus, understanding the cultural context of IU is essential for developing more effective, culturally sensitive interventions. CBT, when adapted to meet the cultural values and expectations of different populations, can address the unique ways in which uncertainty is perceived and managed, improving treatment outcomes for individuals from diverse cultural backgrounds.

In conclusion, demographic factors, such as age, gender, and culture, play a critical role in shaping the experience and management of IU. Tailoring therapeutic approaches to these demographic differences can enhance the effectiveness of treatments for IU and related disorders. Future research should continue to explore these demographic variations to develop more personalized and effective interventions.

## Conclusions

People worldwide are facing highly volatile, unpredictable, and mutually reinforcing environmental and sociopolitical stressors, which act as a massive source of uncertainty, particularly when amplified by media news traversing various media outlets and social media platforms. As reviewed earlier, sufficient evidence now links uncertainty-inducing media news to negative mental health outcomes at the individual level. Moreover, the defensive needs to alleviate uncertainty are linked to maladaptive behaviors, with wider negative societal consequences. Therefore, resilience building against uncertainties originating in a highly volatile, unpredictable, and threatening external sociopolitical environment should be seen as a significant challenge for democratic societies. Effective prevention and remediation strategies for media-induced uncertainty stress require a multilevel approach that integrates adaptive media consumption, cognitive restructuring, and neuroplasticity-informed resilience training. Given the increasing prevalence of global crises and uncertainty-inducing media narratives, interventions aimed at enhancing cognitive flexibility, promoting mindfulness, and developing critical media literacy are essential for mitigating the negative psychological consequences of excessive media exposure. Future research should address the identified research gaps in the following ways:

- Expand study designs while prioritizing longitudinal studies to track changes in IU over time, especially during prolonged crises (eg, pandemics and wars)
- Improve measurement validity by standardizing IU assessment tools across cultures and crises to improve the comparability of findings
- Focus on cross-cultural comparisons and explore regional differences in how media portrays uncertainty and how populations psychologically respond to it
- Investigate media narratives and study how different reporting styles (eg, sensationalized vs neutral reporting) affect uncertainty-related stress in real time, thus clarifying media’s role in shaping psychological outcomes.

## Abbreviations

ACT: acceptance and commitment therapy
CBT: cognitive behavioral therapy
CBT-IU: cognitive behavioral therapy for intolerance of uncertainty
DBT: dialectical behavior therapy
IU: intolerance of uncertainty
MBI: mindfulness-based intervention
PTSD: posttraumatic stress disorder
